# Impact of neuroimaging in the pretreatment evaluation of early stage non-small cell lung cancer

**DOI:** 10.1016/j.heliyon.2020.e04319

**Published:** 2020-06-29

**Authors:** Garrett T. Wasp, Christopher Del Prete, Jonathan A.D. Farrell, Konstantin H. Dragnev, Gregory Russo, Graham T. Atkins, Joseph D. Phillips, Gabriel A. Brooks

**Affiliations:** a Department of Internal Medicine, Section of Medical Oncology, Dartmouth-Hitchcock Medical Center, 1 Medical Center Dr, Lebanon, NH, 03765, USA; b Department of Medicine, Division of Hematology/Oncology, Warren Alpert School of Medicine at Brown University, 222 Richmond St, Providence, RI, 02903, USA; c Department of Radiology, Dartmouth-Hitchcock Medical Center, USA; d Section of Radiation Oncology, Dartmouth-Hitchcock Medical Center, USA; e Department of Internal Medicine, Section of Pulmonology, Dartmouth-Hitchcock Medical Center, USA; f Department of Surgery, Section of Thoracic Surgery, Dartmouth-Hitchcock Medical Center, USA

**Keywords:** Health sciences, Oncology, Medical imaging, Radiology, Diagnostics, Non-small cell lung cancer, MRI brain, Pretreatment evaluation, CT head, Staging, Outcomes

## Abstract

**Background:**

There are limited data and conflicting guideline recommendations regarding the role of neuroimaging in the pretreatment evaluation of non-small cell lung cancer (NSCLC).

**Methods:**

We performed a retrospective, pragmatic cohort study of patients with NSCLC diagnosed between January 1 and December 31, 2015. Eligible patients were identified from an institutional tumor registry. We collected all records of pretreatment neuroimaging within 12 weeks of diagnosis, including CT head (CT) and MRI brain (MRI). We abstracted the indication for neuroimaging, presence of central neurologic symptoms and cancer stage (with and without neuroimaging findings) from the tumor registry and the electronic health record.

**Results:**

We identified 216 evaluable patients with newly diagnosed NSCLC. 157 of 216 patients (72.7%) underwent neuroimaging as part of initial staging, and 41 (26%) were found to have brain metastases. Of 43 patients with central neurologic symptoms at the time of neuroimaging, 28 (67%) had brain metastasis. In patients without central neurologic symptoms, brain metastases were discovered in 0 of 33 patients with clinical stage I or II, 4 of 36 (11%) with clinical stage III and 9 of 45 (20%) with clinical stage IV disease.

**Conclusions:**

In patients with early stage NSCLC (i.e. clinical stage I and II) without central neurologic symptoms, brain metastases are unlikely. The continued use of neuroimaging in the pretreatment evaluation of clinical stage I patients without central neurologic symptoms is not needed.

## 1 Introduction

Accurate staging is essential for determining the appropriate treatment of non-small cell lung cancer (NSCLC). While the role of neuroimaging for locally advanced or neurologically symptomatic NSCLC is firmly established, the role of performing neuroimaging routinely in early stage NSCLC patients without central neurologic symptoms (clinical stage I or II) is not known. Uncertainty regarding the role of neuroimaging in this setting can be seen in the variable recommendations for the pretreatment evaluation of NSCLC. The 2013 American College of Chest Physicians (ACCP) Guidelines on the Diagnosis and Management of Lung Cancer recommend brain MRI or head CT in the pre-treatment evaluation of stage III-IV disease, regardless of the presence of neurologic symptoms [1]. The National Cancer Comprehensive Network (NCCN) Guidelines 2019 recommend brain MRI or CT in stage II-IV disease, and consider it optional in stage IB disease [2]. The routine use of neuroimaging in the pretreatment evaluation of all patients with NSCLC inevitably contributes to delays in treatment initiation and adds to patient burden. Moreover, neuroimaging comprises almost one-third of the cost of the pretreatment evaluation of NSCLC [3]. Identifying subgroups of patients for whom neuroimaging may be unnecessary offers the potential to decrease both financial and patient hardships, e.g. fewer diagnostic tests and less delay in treatment.

A key factor in determining the value of pretreatment neuroimaging in early stage NSCLC is the accurate estimation of the prevalence of brain metastasis in this group. Literature-based estimates of the prevalence of brain metastasis in this subgroup are variable, ranging from 1% to 20% [1, 3, 4, 5, 6, 7]. Many potential reasons for this wide variation have been offered, including inherent differences in populations screened, changes in imaging technology over time, and different TNM staging definitions. For example, asymptomatic brain metastasis were found in only 1.3% of patients from a large, recent cohort of surgically-treated NSCLC patients [3]. Another large cohort study involving both surgical and nonsurgical patients, but restricted to squamous histology, found 0 of 121 stage I patients (0%) and 4 of 125 stage II patients (3.5%) had neurologically asymptomatic brain metastases in the pretreatment period [8].

By reviewing the pretreatment evaluation of patients with pathologically confirmed NSCLC in a calendar year captured in our rural, academic health system, we analyzed the use and findings of neuroimaging in the pretreatment evaluation of all patients with newly diagnosed NSCLC. Specifically, we sought to provide an answer to how frequently neuroimaging identified brain metastasis across different stages of NSCLC, with special attention to common clinical situations where we suspected low and high clinical value.

## 2 Methods

### 2.1 Study design

We performed a retrospective, pragmatic cohort study of patients with NSCLC diagnosed between January 1 and December 31, 2015. We used our health system's tumor registry to identify all cases of biopsy-confirmed non-small cell lung cancer (NSCLC) identified in the study period. All subtypes of adenocarcinoma and squamous cell carcinoma of the lung were included [9]. Exclusion criteria were 1) any prior history of lung cancer, 2) a second cancer with either diagnosis or active treatment within 12 months of NSCLC diagnosis, 3) uncertainty regarding whether the lung was the primary site of malignancy, and 4) incomplete records from the pretreatment staging evaluation. This study was approved by the Dartmouth College Institutional Review Board.

### 2.2 Data collection

We abstracted clinical data, including findings from the pretreatment staging evaluation, from the institutional electronic medical record. All staging determinations used the American Joint Committee on Cancer/Union for International Cancer Control TNM staging manual, Seventh Edition [10]. The clinical stage was derived from results of diagnostic tests including CT, PET/CT, MRI and pathology testing (e.g. fluid cytology, CT guided biopsy). The clinical stage excluding neuroimaging was derived using the same diagnostic tests, except the results of CT head and MRI brain were excluded (regardless of where in the diagnostic sequence these tests were performed). Diagnostic studies that were performed after neuroimaging but before the start of anticancer therapy (lung resection surgery, radiation or systemic anticancer therapy) were included. In instances where diagnostic studies revealed equivocal findings of possible extrathoracic metastatic sites that were not pathologically confirmed, the treating provider's clinical impression was used, as recorded in the medical record. In contrast, when multiple, malignant-appearing lung nodules/masses were identified, they were considered the same malignancy (e.g. higher stage) rather than separate primaries even if treating providers viewed them as simultaneous primaries. Treatment modalities used were included in the tumor registry data, but were also independently verified in the medical record. Staging data reported in the tumor registry were confirmed by review of the findings in the electronic medical record, and when discrepancies persisted after a second review, the reviewers' staging assessment was used. A single reviewer abstracted data for each patient record.

To be included in the pretreatment evaluation, neuroimaging studies had to have been obtained no more than 12 weeks from the initial diagnostic biopsy and before the start of antineoplastic treatment (inclusive of radiation therapy, surgery, or chemotherapy). Any neuroimaging obtained after the start of antineoplastic treatment was excluded. Primary radiology reports (CT, PET/CT, MRI) were reviewed when available. When radiology reports were unavailable, the treating provider's note was reviewed for a summary description of the pertinent radiographic findings. If no summary of CT body imaging, PET/CT or neuroimaging was available, and the study was documented to have occurred, then those charts were excluded from our analysis. Imaging studies were considered “not performed” when both of the following criteria were met: 1) no imaging report existed in our system in the specified pretreatment period AND 2) no mention of the CT, MRI or nuclear study in the treating providers' notes.

For each patient who received pretreatment neuroimaging, we classified the indication for the neuroimaging study as either “symptomatic” or “asymptomatic”. This classification was determined in two-step process. If the study indication from the radiology report contained any central neurologic symptom (e.g. headaches, lightheadedness, altered mental status, dizziness, vision changes, falls, weakness) or listed a known history of neurologic disease, the indication for neuroimaging was categorized as “symptomatic.” The second step involved reviewing the medical records of those patients without any central neurologic sign/symptom in the study indication for the notes in the two months preceding the neuroimaging. These notes were specifically reviewed for any mention of a central neurological symptom or sign in the history of present illness, review of systems, physical exam or assessment and plan. If a central neurologic symptom or sign was documented in the chart, then the patient was categorized as “neurologically symptomatic.” Only if the neuroimaging indication and provider notes show no central neurologic symptoms/signs was the indication categorized as “asymptomatic.” We also recorded the specialty of the physician ordering neuroimaging, and the dates of neuroimaging, diagnostic biopsy and PET/CT.

### 2.3 Neuroimaging

Since the study design was pragmatic (e.g. real-world), we exclusively used the patients’ radiology reports to indicate the presence or absence of brain metastases. There was no independent review of the neuroimaging. The majority of neuroimaging studies (>75%) were performed at the main campus of the academic medical center; the remainder were obtained outside this health system and we are unable to confirm details of imaging technique.

For the studies performed at our center, all brain MRI and CT studies were interpreted by neuroradiology sub-specialty trained, board certified radiologists as part of routine clinical practice at our academic medical center. The MRI protocol for the evaluation of brain metastases includes the following sequences: axial and sagittal T1 pre-contrast, axial diffusion weighted (b = 1000 s/mm^2^), axial T2 fast spin echo, axial FLAIR, axial T1 weighted post contrast, coronal T1 weighted fat-saturated post contrast, and high resolution 1.5mm 3D volumetric T1 weighted post contrast images. Head CT (with or without iodinated contrast) are acquired with helical acquisition from the top of the head to the base of the skull. Images are reconstructed with 1.25mm and 5mm axial slice thickness with standard soft tissue algorithm. Additional thin 1.25mm axial reformatted images are generated with bone algorithm. Coronal and sagittal reformats are generated at 3mm slice thickness.

### 2.4 Analysis

Among patients who had neuroimaging, we report the proportion who were found to have brain metastases. Subgroup analyses were defined first by the presence of central neurologic symptoms, and second by the clinical stage excluding neuroimaging among patients without central neurologic symptoms. Proportions are reported with point estimates. For key subgroups, we also calculated 95% confidence intervals, using a one-sample test of proportions. We identified patients without central neurologic symptoms and Stage I and II disease as a key analytic subgroup of interest.

## 3 Results

**Figure 1:**
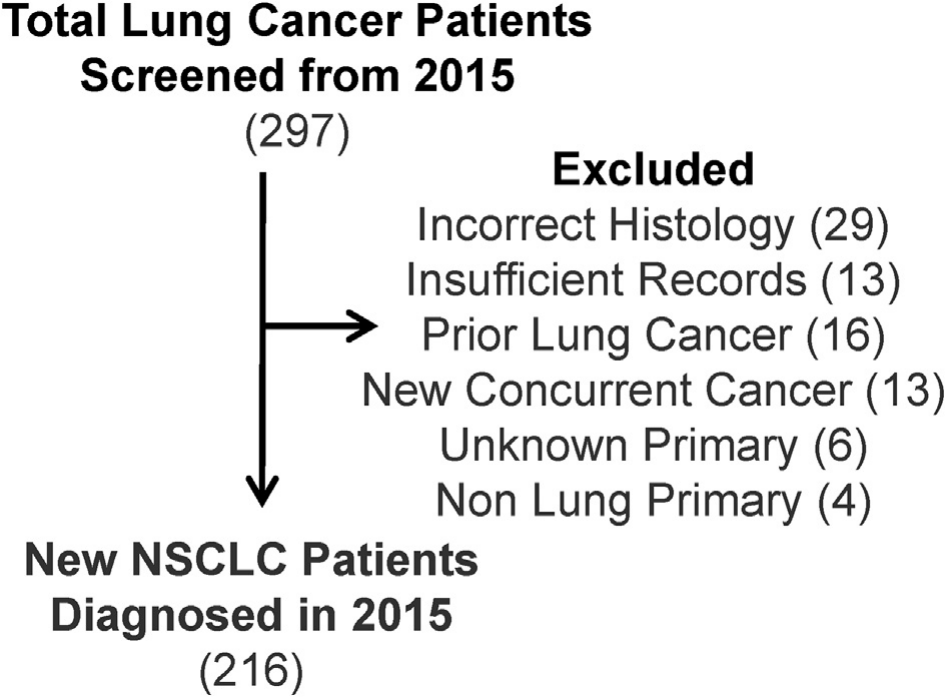
Study patient flow diagram.

Of 297 patients initially screened for inclusion, we identified 216 patients with a new diagnosis of NSCLC meeting inclusion criteria (Figure 1). Cohort demographics for the 216 included patients and the subset of 157 patients (73%) who also underwent neuroimaging are shown in Table 1. 59 patients did not undergo neuroimaging, and most were stage I: 44 patients (stage I), 2 patients (stage II), 5 patients (stage III), and 8 patients (stage IV) as shown in Table 2.

**Table 1:** Characteristics of patients with non-small cell lung cancer.

	All Patients	Patients with neuroimaging
Cohort Size	216	157
Median Age ±SD, y	73 ± 3.1	72 ± 4.2
Sex, n (%)		
Male	112 (52)	83 (53)
Female	104 (48)	74 (47)
Tumor histologic type, n (%)		
Adenocarcinoma	140 (65)	99 (63)
Squamous	72 (33)	54 (34)
NOS	4 (2)	4 (3)
Clinical Stage, n (%)		
I	69 (32)	24 (15)
II	18 (8)	15 (10)
IIIA	28 (13)	26 (17)
IIIB	9 (4)	7 (4)
IV	92 (43)	84 (54)
Treatment modalities, n (%)		
Chemotherapy alone	34 (16)	29 (18)
Radiation alone	25 (12)	15 (10)
Surgery alone	45 (21)	18 (11)
Chemotherapy + radiation	64 (30)	60 (38)
Chemotherapy + surgery	20 (9)	11 (7)
Radiation + surgery	2 (1)	2 (1)
Chemotherapy, radiation and surgery	9 (4)	9 (6)
No anticancer treatment	17 (8)	13 (8)

**Table 2:** Neuroimaging findings stratified by clinical stage^†^ and presence of central neurologic symptoms.

Clinical Stage^†^ (N)	CNS symptoms? N (% previous category)	Imaging? N (% previous category)	Brain Metastasis? N (row %)
I (71)	Yes 10 (14)	Yes 10 (100) ((1(100)	3 (30)
		No 0	-
	No 61 (86)	Yes 17 (28)^‡^	0
		No 44 (72)	-
II (20)	Yes 2 (10)	Yes 2 (100)	2 (100)
		No 0	-
	No 18 (90)	Yes 16 (89)	0
		No 2 (11)	-
III (48)	Yes 7 (15)	Yes 7 (100)	6 (86)
		No, 0	-
	No 41 (85)	Yes 36 (88)	4 (11)
		No 5 (12)	
IV (77)	Yes 24 (31)	Yes 24 (100)	17 (71)
		No 0	-
	No 53 (69)	Yes 45 (85)	9 (20)
		No 8 (15)	-

Central neurologic symptoms (CNS).

†Clinical Stage that excluded neuroimaging.

‡Eight (8) patients in this group had cIA disease. There were 48 patients in total with cIA disease, therefore 8/44 (18%) cIA underwent neuroimaging.

### 3.1 Prevalence of brain metastases

Of all patients who had pretreatment neuroimaging, 41 of 157 (26%) had a finding of brain metastasis. Central neurologic symptoms were present at baseline in 43 patients and 28 of 43 symptomatic patients (67%) were found to have brain metastases. Among 114 patients without central neurologic symptoms at the time of neuroimaging, 13 of 114 patients (11%) were found to have brain metastases. After stratifying this asymptomatic population by clinical stage exclusive of neuroimaging results, brain metastases were found in 0 of 17 patients (95% CI: 0–19.5%, stage I), 0 of 16 patients (95% CI: 0–20.6%, stage II), 4 of 36 patients (11%, 95% CI: 3.1–26.1%, stage III), and 9 of 45 patients (20%, 95% CI: 9.6–34.6%, stage IV) as shown in Table 2.

### 3.2 Ordering and sequencing of neuroimaging services

Review of the neuroimaging studies in our study population demonstrated that 74% of all patients with newly diagnosed NSCLC had neuroimaging as part of their pretreatment evaluation (144 MRIs and 13 CT heads alone).73% of neuroimaging was performed in patients without clinically noted central neurologic symptoms. The majority of neuroimaging was performed as an outpatient (78%). Most of the neuroimaging studies (41%) were ordered by pulmonologists, followed by internal/family medicine (20%), oncology (15%), surgery (12%), other (10%), unknown specialty (1%) and radiation oncology (1%) as shown in Table 3.

**Table 3:** Neuroimaging modalities and specialty of physicians ordering neuroimaging.

	All Imaged (n = 157)	All Stages, w/o CNS symptoms (n = 114)	Stage I and II w/o CNS symptoms (n = 33)
**Neuroimaging testing**, **n (% column)**			
CT head	13 (8)	10 (9)	2 (6)
MRI brain	144 (92)	104 (91)	31 (94)
MRI and CT head	23 (15)	2 (2)	0
**Site of Testing, n (% column)**			
Outpatient	123 (78)	103 (90)	32 (97)
Inpatient	34 (22)	11 (10)	1 (3)
**Documented CNS symptoms? n (% column)**			
Yes	43 (27)	-	-
No	114 (73)	-	-
**Ordering Physician Department, n (% column)**			
Pulmonology	64 (41)	59 (52)	16 (48)
Internal/Family Medicine	31 (20)	19 (17)	4 (12)
Oncology	24 (15)	20 (18)	6 (18)
Surgery	19 (12)	14 (12)	7 (21)
Other	16 (10)	1 (1)	0
Unknown	2 (1)	0	0
Radiation Oncology	1 (1)	1	0

We also evaluated the sequencing of the three major diagnostic procedures (biopsy, PET/CT and neuroimaging) for the subgroup of neurologically asymptomatic patients with stage I and II NSCLC (33 patients). We found 13 patients (39%) had neuroimaging as the last major diagnostic test, 11 patients (33%) had neuroimaging and PET/CT last (on the same day), and the remaining 9 patients (27%) had either PET/CT or biopsy as their last major diagnostic study. In the group of 13 patients where neuroimaging was the last major diagnostic study, the neuroimaging study occurred a median of 17 days (range 3–51 days) after the most recent previous major diagnostic study.

## 4 Discussion

The prevalence of brain metastasis among patients with newly-diagnosed NSCLC is poorly defined, and studies examining this question have offered estimates ranging from 1-20% [1, 3, 4, 5, 6, 7]. This wide range of uncertainty in the prevalence of asymptomatic brain metastasis has resulted in conflicting recommendations from medical societies for neuroimaging in patients with clinical stage I/II disease. It is also likely that differing opinions regarding what constitutes an acceptable false negative rate (e.g. failing to detect a brain metastasis) contribute to the lack of consensus regarding diagnostic testing in this population. While more recent estimates have suggested that the prevalence of brain metastasis at diagnosis in stage I or II patients is low (<1% for Stage I and <4% Stage II) [3, 8], these estimates come from selected patient populations (surgical patients, patients with squamous cell lung cancer) making it problematic to generalize the findings to the broader population of patients with a new diagnosis of NSCLC. In our population of surgical and non-surgical patients with NSCLC, we found that neuroimaging had a low yield (0%) for detecting brain metastasis in patients with stage I or II disease without central neurologic symptoms. Furthermore, we found that neuroimaging delayed the completion of diagnostic testing for 39% of patients in this subgroup, with a median delay of 17 days from the last major diagnostic test. This finding is significant because delays in time to treatment have been shown to be a source of significant anxiety in this population [11]. In contrast, the presence of central neurologic symptoms (67% of these patients identified as having brain metastasis), or higher stage disease without central neurologic symptoms (11% for stage III and 20% for stage IV disease found to have brain metastasis) were associated with higher detection rates of brain metastasis.

Our data on the prevalence of brain metastasis in the initial presentation of NSCLC patients correspond well with the findings in more recent literature. A Korean single institution cohort of surgical and nonsurgical patients from 2012-2013, who were stratified by central neurologic symptoms and clinical stage excluding neuroimaging, found brain metastases in 0/121 (0%) stage I patients and 4/135 (3.0%) stage II patients; however, their cohort included only squamous cell histology [8]. We also know from cohorts of NSCLC patients with asymptomatic brain metastasis, some of these patients had T1-2a N0 disease, meaning that the true incidence of brain metastasis in Stage I patients is not 0% [12]. A population study using cancer registry data from Canadian and American patients with NSCLC diagnosed in 2010–2011 found an overall prevalence of brain metastasis from lung cancer of 10% [13], but they did not stratify findings by clinical stage or central neurologic symptoms. In the medical literature review underpinning the ACCP's recommendation to obtain routine imaging of the brain in NSCLC patients with stage III or IV disease, the authors found nine studies that reported the prevalence of brain metastasis in patients with negative clinical evaluation for central neurologic symptoms, and the median prevalence was 3% [1, 14]. The studies in this group that reported a prevalence of 5% or greater for stage I or II NSCLC were older studies that used different editions of the AJCC staging manual, did not include PET/CT, and some included histology types of lung cancer that are now classified after the WHO 2015 revision under neuroendocrine carcinoma (e.g. large cell carcinoma) [15, 16, 17, 18, 19, 20, 21, 22, 23]. Lastly, the rate of routine neuroimaging we report in Stage Ia patients (18%, 8 of 44 patients) is comparable to the overall rate found in the multicenter National Lung Screening Trial (NLST) (12%, 77 of 643) [24]. Albeit this pattern of neuroimaging use predated the 2013 Choosing Wisely recommendation since data collection for NLST occurred from 2002-2009 [25].

Our data helps support the ACCP 2013 guideline to omit neuroimaging in neurologically asymptomatic, early stage NSCLC [1]. Contextualizing our findings with the work of Lee et al 2016, the use of routine neuroimaging in Stage I patients without central neurologic symptoms is likely to be of low value and should be avoided [8]. The routine use of neuroimaging in stage II patients without central neurologic symptoms needs further investigation to better refine the estimated prevalence of brain metastasis in this population. Additionally, if the true prevalence of brain metastasis in Stage II NSCLC patients is somewhere between 2-5%, the community of physicians managing NSCLC will need to reflect on the benefits of more accurate staging for this limited group weighed against the added patient and financial costs of many more.

We also confirmed that despite the 2013 Choosing Wisely® recommendations of the Society of Thoracic Surgeons discouraging the use of neuroimaging in Stage IA disease, this practice is still occurring. Our study has important implications for those who are interested in decreasing the number of potentially low value neuroimaging studies ordered in the pretreatment period for NSCLC. The management of NSCLC often involves multiple specialties and therefore it is not obvious who is guiding the diagnostic workup. At our rural, academic health network, we found that the specialties involved in the definitive treatment of the cancer (radiation oncology, surgery and medical oncology) were responsible for only 28% of the neuroimaging orders overall, and 39% of the neuroimaging studies in our prespecified sub-group of interest (Stage I and II patients without central neurologic symptoms). Another important insight is that there appears to be more than one pattern of ordering behavior for neuroimaging at work for our cohort of interest. This can be inferred from the variability seen in the sequencing of the three major diagnostic studies (PET/CT, Neuroimaging, and Biopsy). Since PET/CT is a strong determinant of clinical stage, the fact that neuroimaging and PET/CT occurred on the same day for 33% of stage I and II patients without central neurologic symptoms, signals that for these same-day patients, ordering physicians are not making a stepwise decision (e.g. if stage II, III or IV disease, then obtain MRI), rather it is more likely treated as bundle (e.g. has NSCLC, obtain PET/CT and neuroimaging). Conversely, we suggest that neuroimaging that is happening after PET/CT, especially when it several days or weeks later, reflects a physician making a decision about obtaining neuroimaging after having a more accurate stage assessment (e.g. patient likely has stage II NSCLC, obtain neuroimaging). Our results suggest that decision support interventions should target three specialties (pulmonology, medical oncology, and surgery) since they account for almost 90% of the imaging in this subgroup and recognize that different patterns of ordering are at work.

The limitations of our study primarily stem from the study design of a single institution, retrospective cohort with small numbers in some patient subgroups. We attempted to account for referral center bias by including patients treated at our affiliated cancer centers spread across our geographic region, which represents an estimated service area of 16,000 square miles. Nevertheless, there are oncology providers outside our academic health system who also practice in the same geographic area. As such, patient selection bias could exist. Given the design, we cannot know the prevalence of brain metastasis in early stage NSCLC patients who did not undergo neuroimaging. However, we do not have reason to suspect that it would be significantly different from what is presented above. Our methodology was reliant upon documentation within the medical record, which makes our study potentially susceptible to three issues. The first reflects that the absence of documentation of an imaging study does not necessarily mean the study was not performed. However, given the importance of imaging in the pretreatment evaluation and decision-making for cancer treatment, we feel it likely that treating providers would be diligent in recording a staging study when it occurred outside our system, especially since patients are presented to colleagues at a common thoracic tumor board. In a related concern, documentation alone cannot tell whether a patient is truly neurologically asymptomatic from a brain metastasis. However, it is a good surrogate for gauging the provider's awareness of central neurologic symptoms, which is important since we are ultimately interested in the physician's intent in ordering the neuroimaging study. Thirdly, the documented “MRI ordering physician department” data point may not fully capture the physician behavior around obtaining neuroimaging. Specifically, physicians who do not routinely evaluate patients for lung cancer (e.g. internal medicine and family medicine) may ask a colleague who is more familiar, or they could be required to order a study as a precondition for making a referral. The effect on our measure would be to overestimate the contribution of internal and family medicine doctors as ordering department for neuroimaging studies in this cohort of NSCLC patients. Lastly, we chose a pragmatic design so while independent radiographic review could have altered the measured rates of brain metastasis, our approach is more consistent with routine clinical care where a single radiologist reviews neuroimaging.

In conclusion, our results do not support the routine use of neuroimaging in the pretreatment evaluation of clinical stage I patients without central neurologic symptoms.

## Declarations

### Author contribution statement

G. Wasp and C. Del Prete: Conceived and designed the experiments; Performed the experiments; Analyzed and interpreted the data; Wrote the paper.

J. Farrell and G. Brooks: Conceived and designed the experiments; Analyzed and interpreted the data; Wrote the paper.

K. Dragnev, G. Russo, G. Atkins and J. Phillips: Analyzed and interpreted the data; Wrote the paper.

### Funding statement

This work was supported by 10.13039/100008300The Dartmouth Clinical and Translational Science Institute, under award number UL1TR001086 from the 10.13039/100006108National Center for Advancing Translational Sciences (NCATS) of the 10.13039/100000002National Institutes of Health (NIH), and the 10.13039/100009230Norris Cotton Cancer Center for open access.

### Competing interest statement

The authors declare no conflict of interest.

### Additional information

No additional information is available for this paper.
